# Epidemiological Study of Equine Piroplasmosis (*Theileria equi* and *Babesia caballi*) by Microscopic Examination and Competitive-ELISA in Erbil Province North-Iraq

**Published:** 2019

**Authors:** Khalid Jabar AZIZ, Lokman Taib Omer AL-BARWARY

**Affiliations:** 1. Department of Animal Resources, College of Agricultural Engineering Sciences, Salahaddin University-Erbil, Erbil, Iraq; 2. Department of Microbiology and Pathology, College of Veterinary Medicine, University of Duhok, Duhok, Iraq

**Keywords:** Epidemiology, Equine piroplasmosis, Erbil

## Abstract

**Background:**

Equine piroplasmosis is a major tick-borne disease that can lead to serious health problems and economic losses in horse industry. The aim of the study is to determine the prevalence of *Theileria equi* and *Babesia caballi* in different species of Equus namely (Horse, mule, donkey and pony) by Giemsa stained blood films and competitive ELISA.

**Methods:**

This study was conducted at various geographic areas of Erbil governorate. A total of 349 blood samples from equine (209 horses, 62 mule, 57 donkey and 21 ponies) were collected to estimate the prevalence rate of *Theileria equi* and *Babesia caballi* by using Giemsa stained blood smear and competitive ELISA.

**Results:**

The overall prevalence rates were (10.6%) consisting of (8.3%), (1.7%) and (0.6) for *T. equi*, *B. caballi* and both infection respectively by Giemsa stained blood smears, and the rate was 38.97%, consisting of 20.9% for *T. equi*, 11.2% for *B. caballi* and 6.9% for both infection by cELISA. Seroprevalence rate of *T. equi* was significantly higher (*P* < 0.001) than that *B. caballi* in equids. There was also a significant difference associated between age (*P* < 0.01), activity (*P* < 0.01), management (*P* < 0.001) and tick infestation (*P* < 0.001), but neither the type of equids nor the gender was significant differences associated with prevalence rate.

**Conclusion:**

The present study concluded that the equine piroplasmosis is a vital infection distributed among the equine in Erbil province North of Iraq. Thus a better control programme should be implemented to contain and control the prevalence of the disease within the area.

## Introduction

Equine piroplasmosis is one of the most common tick-borne hemoprotozoan diseases that poses serious threats to the equids including horses, ponies, mules, donkeys and zebras, has important implications for the international movement of horses (1). The disease is caused by either *Theileria equi* or *Babesia caballi* or both protozoa (2). These blood protozoa are principally transmitted by the tick of ixodid family (3).

Equine piroplasmosis can cause significant economic losses that include the cost of treatment decreased equids production, still birth, lack of performance or death and inability to meet international requirements for exports or involvements in equestrian sports (4). Also, some countries do not allow the entry of subclinical seropositive horses (5). The clinical manifestations of the disease ranging from subclinical to acute potentially fatal infection depend on the equide immune status and the virulence of protozoa. The clinical signs characterised by fever, anaemia, icteric sclera, hemorrhagic mucus membrane, weakness, lethargy, dark urine, anorexia and mild colic with the reduced fecal output (6). However, most of the equine that recovers from infection are become carriers for several years and reservoirs for vector ticks (7, 8). Identification of carrier equines is essential for the evaluation of infection risk as they serve as a pool of infection for ticks and cause natural transmission of the disease (9). In the epidemiological studies in different countries, there are many risk factors related with the high prevalence of equine piroplasmosis such as equids species, age, gender, breed, the presence of tick, activity and regions (10, 11).

Several methods can diagnose equine piroplasmosis, direct microscopic identification of the parasite in stained Giemsa blood smears is confirmatory only during the acute stage of the infection, but it seems to be unsatisfactory to diagnoses in carrier animal, and it is also not practical on a large scale (7). Therefore, serological tests are recommended as a select method of diagnosis during latent stage of infection, especially when horses designed to be imported into regions that are free from the disease, while the tick vectors are present (12). In 2004, the OIE approved the competitive inhibition enzyme-linked immunosorbent assay (cELISA) for detection of antibodies against *T. equi* and *B. caballi*, and as a specified test for global horse activity (13). Later, direct detection of these haemoprtozoan infection can be carried out using molecular tools such as conventional PCR (14), multiplex PCR (15), nested PCR (16), real-time PCR (17) and reverse line bloat (RLB) hybridization assay (18) have been developed and are reliable diagnostic tools.

There is no previous serological study concerning the occurrence of *T equi* and *B. caballi* infection in equine in Erbil governorate, north of, Iraq. Therefore, the objective of this research is firstly to determine the prevalence of *T. equi* and *B. caballi* in equine by Giemsa stained blood smears and secondly to determine the presence of anti-*Theileria equi* and anti-*Babesia caballi* antibodies using cELISA.

## Materials and Methods

### Sample collection and study design

This study was carried out under the supervision and regulations of Ethic Committee at College of Veterinary medicine, University of Duhok, Iraq.

A total of 349 blood samples from equine (209 horses, 62 mule, 57 donkey and 21 ponies) were collected via jugular vein using anti-coagulant and without any anti-coagulant sterile vacutainers^®^ tubes. They were sampled in various geographic areas of Erbil governorate. The sex, age and breed of the equine were also recorded during this study.

### Examination of blood smears

Thin blood smears were prepared from blood samples in EDTA tubes. The smears were then air dried, fixed in absolute methanol for 5 minute and stained with 10% Giemsa stained for 30 minute to determine the parasite in erythrocytes. The slides were examined with an oil immersion lens at magnification of ×1000 (19).

### Serological detection of *T. equi* and *B. caballi* antibodies

All sera collected from Equine were assessed for the existence of antibodies to *T. equi* and *B. caballi* using a commercial cELISA test kit (VMRD, Inc., Pullman, and WA99163 USA) methods following the manufacturer's instructions. This serological analysis identify serum antibodies against Equine Merozoite Antigen 1 (EMA-1) surface protein of *T. equi* (20), and rhoptry-associated protein (RAP-1) of *B. caballi* (21). The optical density (OD) values of the controls and samples were measured at 630 nm wave length using an automatic UV max kinetic microplate reader (BioTek^®^ Elx800, USA), and the percentage of inhibition (%) was calculated as follows: I (%)=100-(sample OD ×100)/(mean OD of three negative controls). Serum samples with ≥40% inhibition were considered positive and samples with <40% inhibition were considered negative as recommended by the manufacturer.

### Statistical analysis

The *χ*^2^ and Fischer's exact test were used to differentiate the prevalence of equine piroplasmosis between various groups. Binomial logistic regression in GenStat 12^th^ Edition was used to determine odds ratio and the effect of risk factors (type of equine, gender, age of group, purpose of keeping and whether tick is found on equine or not) on the incidence of *T. equi, B. caballi* and both protozoa. All candidate variables were kept in the model with significant attributes at *P* <0.05.

## Results

### Prevalence of equine pirolasmosis (EP)

**Fig. 1:**
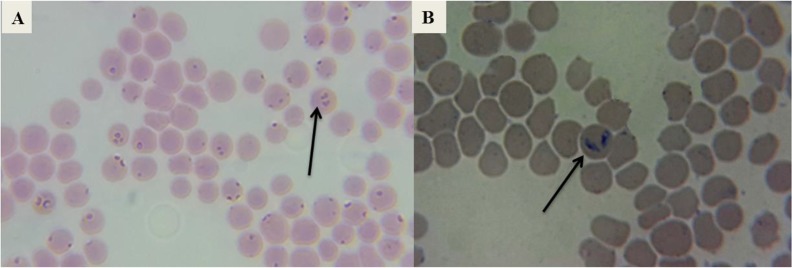
Blood smear stained with Giemsa showed, A) several blood cells infected with *T.equi* which appear as pyriform (a pair of joints) and single pyriform. B) *B. caballi* appear as double pear acute and an obtuse angle, single pear and round shape (at magnification of ×1000)

Both protozoa morphological evident in stained smears, the polymorphic shapes of *T. equi*, oval, rod, single pear and double pear were observed in erythrocyte as in Fig. 1-A, and *B. caballi* appeared as single pyriform or double pear and an obtuse angle was observed as in Fig. 1-B. The rate of infection was 6 (1.7%), 29 (8.3%) of *B. caballi* and *T. equi* respectively, the mixed infection rate was 2 (0.6%) by Giemsa stained blood smears (Table 1).

**Table 1:** Prevalence of *Theileria equi*, *Babesia caballi* and both protozoa in equids by microscopic and serological examination

***Type of protozoa***	***No. of equine tested***	***Microscopic examination***			***cELISA***		
*B. caballi*		6 (1.7)	1.00		39 (11.2)	1.00	*P*
*T. equi*	349	29 (8.3)	5.18 (2.12–12.64)	<0.001	73 (20.9)	2.1 (1.38–3.20)	<0.001
Both protozoa		2 (0.6)	0.33 (0.07–1.61)	0.17	24 (6.9)	0.59 (0.34–0.99)	0.05
Overall		37 (10.6)			136 (38.9)	21.8 (76)	

N: number of positive samples

The overall seroprevalence of EP in this study by cELISA was about 38.9% (136/349) (Table 1). Regarding the type of protozoa, this study found that the seroprevalence was 11.2% (39/349) and 20.9% (73/349) for *B. caballi* and *T. equi*, respectively. This work also found that the prevalence rate of mixed infection was 6.9% (24/349); the seroprevalence rate of *T. equi* was proximately two times more than *B. caballi* (Table 1). The cELISA has significant advantage than microscopic detection because of greater sensitivity and specificity for diagnosis of EP.

### Multivariable analysis for the seroprevalence of EP

Four variable conditions were analysed in this seroprevalence study including the type of equids, gender, age and purpose of keeping (Table 2). In the current research, the prevalence of *T. equi, B. caballi* and both protozoa did not significantly differ between types of equids. The seroprevalence of *B. caballi* was significant higher (*P*=0.04) in female (15.3%) than male (7.7%) while no significant differences regarding the seroprevalence of *T. equi* and both infection were detected between genders. The odds infection of *B. caballi* in an age less than five years was 2.88 times greater than equids of other age (CI, 1.31–6.34) with significant differences (*P*=0.01). Despite that no significant differences respecting the seroprevalence of *T. equi* and both infection were observed in age groups 5–10 and >10 years. It also should be noted that recreation (*P*=0.09) and loading (*P*=0.01) appeared to be only significant influence for the prevalence of mixed infection in this study (Table 2).

**Table 2:** Relative risk of equids factors associated with seropositivity of *T. equi*, *B. caballi* and both protozoa

***Factor***	***No. of equine tested***	***T. equi***			***B. caballi***			***Both protozoa***		
		***N. (%)***	***OR (95%CI)***	***P***	***N. (%)***	***OR (95%CI)***	***P***	***N. (%)***	***OR (95%CI)***	***P***
Type of equine										
Donkey	57	11 (19.3)	1		5 (8.8)	1		3 (5.3)	1	
Horse	209	46 (22)	1.18 (0.56–2.45)	0.66	26 (12.4)	1.47 (0.54–4.03)	0.45	13 (6.2)	1.19 (0.32–4.33)	0.78
Mule	62	13 (20.1)	1.11 (0.45–2.71)	0.82	7 (11.3)	1.32 (0.39–4.43)	0.65	7 (11.3)	2.29 (0.56–9.29)	0.25
Pony	21	3 (14.3)	0.69 (0.17–2.79)	0.61	1 (4.8)	0.52 (0.05–4.69)	0.56	1 (4.8)	0.9 (0.09–9.07)	0.93
Gender										
Female	131	32 (24.4)	1		20 (15.3)	1		6 (4.6)	1	
Male	156	28 (17.1)	0.62 (0.35–1.10)	0.1	12 (7.7)	0.46 (0.27–0.98)	0.04	11 (7)	1.58 (0.57–4.38)	0.38
Age group										
5–10	151	32 (21.2)	1		11 (7.3)	1		8 (5.3)	1	
<5	103	25 (24.3)	1.19 (0.66–2.16)	0.56	19 (18.5)	2.88 (1.31–6.34)	0.01	10 (9.7)	1.92 (0.73–5.04)	1.21
>10	95	16 (16.8)	0.40 (0.39–1.46)	0.75	9 (9.5)	1.33 (0.53–3.34)	0.54	6 (6.3)	1.21 (0.41–3.58)	0.74
Purpose of keeping										
Breeding	52	9 (17.3)	1		10 (19.2)			8 (15.4)	1	
Recreation	92	18 (19.5)	1.16 (0.48--2.81)	0.74	5 (5.4)	0.03 (0.09–0.85)	0.29	6 (6.5)	0.38 (0.13–1.17)	0.09
Racing	86	19 (22)	1.36 (0.56–3.27)	0.49	4 (4.7)	0.01 (0.06--0.69)	0.2	6 (6.9)	0.41 (0.13–1.27)	0.12
loading	119	27 (22.7)	1.4 (0.61–3.23)	0.43	20 (16.8)	0.85 (0.36–1.96)	0.7	4 (3.4)	0.19 (0.06–0.66)	0.01

### Association of seroprevalence of EP with management factors

The results of this study revealed that the seroprevalence analysis about the accurate characteristics of the equine population hypothesised to be related with the occurrence of equine piroplasmosis. The current research has showed that the prevalence of *T. equi, B. caballi* and both protozoa infection was higher among equids mixed with other animals in stable compared to equids kept alone, but there were no significantly differences (Table 3). Outdoor feeding management (grazing), was found to be a significant influence for the presence of *T. equi; B. caballi* and mixed infection (*P*<0.001) (Table 3).

**Table 3:** Relative risk of management and ticks factors associated with seropositivity of *T. equi* and *B. caballi*

***Factors***	***No. of equine tested***	***T. equi***			***B. caballi***			***Both protozoa***		
		***N. (%)***	***OR (95% CI)***	***P***	***N. (%)***	***OR (95% CI)***	***P***	***N. (%)***	***OR (95% CI)***	***P***
Animals in stable										
Mixed with other animals	231	54 (23.4)	1		28 (12.12)	1		18 (7.8)	1	
Only equine	118	19 (16)	0.62 (0.35–1.12)	0.12	11 (9.3)	0.67 (0.32–1.43)	0.3	6 (5)	0.52 (0.19–1.44)	0.21
Management										
In grazing	146	47 (32.2)	1		26 (17.8)	1		16 (10.9)	1	
In stable	203	26 (12.8)	0.31 (0.18–0.53)	<0.001	13 (6.4)	0.32 (0.16–0.64)	0.001	8 (3.9)	0.33 (0.13–0.79)	0.01
Presence of ticks										
Not found	213	26 (12.2)	1		12 (5.6)	1		12 (5.6)	1	
On equine	39	27 (69.3)	14.87 (6.78–32.62)	<0.001	9 (23)	5 (1.95–12.91)	<0.001	3 (7.7)	1.39 (0.37–5.19)	0.62
On animals nearby equine	97	20 (20.6)	1.72 (0.91–3.22)	0.09	18 (18.5)	3.8 (1.76–8.27)	<0.001	9 (9.3)	1.71 (0.69–4.21)	0.24

The odds of infection in equines with tick infestation was 14.87 and 5.03 times higher than equids without tick infestation (CI, 6.78–32.62) and (CI, 1.95–12.91) with significant differences (*P*=0.001) and (*P*=0.001) respectively for *T.equi* and *B.caballi.* On the other hand on nearby animals was significantly higher (*P*=0.09) and (*P*<0.001) for *T.equi* and *B.caballi* respectively compared to equines without infestation.

## Discussion

Equine piroplasmosis is a significant disease that can lead to serious health problems and economic losses. Several studies have been previously done in Kurdistan region, Iraq, but were focused on the domestic animals including cattle, sheep and goat (22, 23). Therefore, the objective of this study is firstly done to evaluate the seroprevalence rate of *T. equi; B. caballi* in equine using microscopic examination and cELISA. This study is also determining the several risk factors related to piroplasmosis in equine in Erbil Government.

In the current study, the rate of infection was 6 (1.7%), 29 (8.3%) and 2 (0.6%) of *B. caballi*, *T. equi* and mixed infection respectively by Giemsa stained blood smears. This low rate of infection occurs due to this method has limited sensitivity and specificity detection especially during latent or carrier stage of infection with low level of parasitemia (24). However this method is still commonly used as a cheapest and fastest method for identification of parasites in acute infected animals (25). The overall seroprevalence of EP was 38.9% for all piroplasms. The rate of infection was 11.2% for *B. caballi* and 20.9% for *T. equi,* while the percentage of mixed infection was 6.9% by cELISA. Our study showed that *T. equi* infections were more commonly observed than *B. caballi*, in agreement with most previous studies from another province of Iraq (26) who found that 81.11% for *T. equi* and 18.88% for *B. caballi* of infection in Mosul Governorate. Furthermore, the seroprevalence rate of infection in Basrah Province, Iraq was 86.58% and 54.39% for *B. caballi* and *T. equi* respectively (27). Similar studies conducted in Iraqi neighboring countries were observed that *T.equi* and *B. caballi* seroprevalence between horses was 51% and 2% respectively in Iran (28), 16.21% and 9.6% respectively in Turkey (29), 97.7% and 40.9% respectively in Oman (30), 10.4% and 7.5% respectively in Saudi Arabia (31), 77.1% and 11.4% respectively in Kuwait (32). The higher prevalence of *T. equi* as illustrated in this study could be described due to that *T. equi* is usually life-long than *B. caballi* which generally remain in 4–5 years (33, 34). Moreover, *T. equi* were more pathogenic than *B. caballi* and more public in the endemic area (7). These differences in the prevalence in different climatic place within a country and among nations may be due to variation in sensitivity of the diagnostic tests employed, differences in number and incidence of tick vectors, activity of equines and the presence and useful of control programs (10).

A study of the risk factors for EP identified in this study is presented in Table 2. In our study, there were no significant differences among the type of equids, gender and breeds these indicate that EP is widespread in Erbil governorate due to that EP are affected all type of equines as same as. Mixed infection was significantly higher in recreation and loading. These results are in agreement with another study (35). This may be due to that a physical stressor that may temporarily the immune system and immune-compromised animals have been shown to be more susceptible to infection compared with their immunocompetent animals (29). However, the odds of female gender being infected with *B. caballi* was approximately two times greater than male gender, which was widely consistent with other findings (36, 37). The explanation is that the immune-suppression caused by stress during the third stage of pregnancy and parturition might be the result of higher protozoan infections in female equids, especially if persistently infected and so increase their chance of exposure to disease (38). The prevalence of *T. equi* was significantly higher among equids infested with ticks compared to equids not infested with ticks. This higher seropositivity of protozoa may be due to the ticks that are known to be their main vectors. These results agree with other studies (10, 11), where the presence of ticks was the risk factor associated with equine piroplasms.

The results showed that the seroprevalence of *T, equi* and *B. caballi* and both protozoa infection was higher two times among equids kept with other animals compared to equids isolated in the stable and away from other animals. The same result was obtained already (39). This may be due to these animals acting as reservoirs for *T. equi* and *B. caballi*, as well as tick vectors (40).

This work revealed that the seroprevalence of all type of protozoa infection was significantly higher among equids that grazed than those kept in a stable. The equids that are grazed are more exposed to external environmental conditions that consistent with other findings (10, 41) who found that equids in grazing lands were a high-risk factor for seropositivity of EP.

## Conclusion

Piroplasmosis in equine seems to be widely distributed in Erbil Province, North of Iraq. Our results revealed that the prevalence of EP was considerably higher among equids in the stable when ticks were found on equids, and nearby animals than equids were no ticks infestation found on equine and nearby animals only. The relatively high seroprevalence rate of equine piroplasmosis observed in our study may be the cause of high tick vector population in Erbil province, which is greatly responsible for the transmission of the disease among animals. These findings may help in planning prevention and control strategies for EP in Erbil north of Iraq. However more studies on molecular characterization of EP with large-scale sampling of both equine and vector tick population are needed to investigate for the whole country to allow better control of EP in Iraq.
